# Human Multi-Organ-on-a-Chip Platforms for Next-Generation Drug Delivery Strategies

**DOI:** 10.7150/thno.130030

**Published:** 2026-04-23

**Authors:** Cosmo S. Mitchell, Hong-Il Yoo, Carolina Gracia Diaz, Jordan J. Green, Deok-Ho Kim

**Affiliations:** 1 Chemistry-Biology Interface Program, Johns Hopkins University, Baltimore, MD 21218, USA.; 2 Department of Anatomy and Neurosciences, Eulji University School of Medicine, Daejeon 34824, Republic of Korea.; 3 Department of Biomedical Engineering, Johns Hopkins University, Baltimore, MD 21205, USA.; 4 GSK, Collegeville, PA 19426, USA.; 5 Translational ImmunoEngineering Center, Johns Hopkins University, Baltimore, MD 21205, USA.; 6 Departments of Materials Science & Engineering, Chemical & Biomolecular Engineering, Neurosurgery, Oncology, and Ophthalmology, Johns Hopkins University, Baltimore, MD 21205, USA.; 7 Center for Microphysiological Systems, Johns Hopkins University, Baltimore, MD 21205, USA.; 8 Department of Medicine, Johns Hopkins University, Baltimore, MD 21205, USA.; 9 Departments of Mechanical Engineering and Neurology, Johns Hopkins University, Baltimore, MD 21205, USA.; 10 Institute of NanoBioTechnology, Johns Hopkins University, Baltimore, MD 21205, USA.

**Keywords:** multi-organ-on-a-chip (MOoC), microphysiological systems (MPS), drug delivery, new approach methodologies (NAMs)

## Abstract

Human multi-organ-on-a-chip (MOoC) platforms have rapidly emerged as next-generation preclinical models for evaluating therapeutic delivery and efficacy. By integrating dynamic perfusion, physiologically relevant microenvironments, and systemic tissue-tissue crosstalk, MOoCs provide unprecedented opportunities to study drug absorption, distribution, metabolism, and toxicity under human-relevant conditions. In this review, we summarize recent progress in MOoC technologies and their application to diverse drug delivery strategies, including stem cell-based carriers, gene and nucleic acid therapies, nanoparticles, extracellular vesicles, and biomaterials. We highlight how these systems overcome key limitations of conventional *in vitro* and animal models, offering more predictive insight into therapeutic distribution and pharmacodynamics. Key technical and biological considerations, including material limitations, physiological scaling, and incorporation of patient-specific components, are discussed in the context of ongoing engineering developments. These platforms enable assessment of organ-specific accumulation, metabolic transformation, and off-target effects that are poorly captured by traditional methods. Finally, we outline future perspectives for MOoCs as translational platforms poised to accelerate drug discovery, reduce clinical attrition, and support the development of precision medicines.

## 1. Introduction

Preclinical drug testing provides valuable information early into the drug development process. *In vitro* and animal models provide insight into pharmacokinetics (PK), pharmacodynamics (PD), and off-target effects of a potential therapeutic. However, despite encouraging results in cellular or animal experiments, translation to human clinical trials remains a critical bottleneck. A vast majority of drug candidates that enter clinical testing fail due to a lack of efficacy or unacceptable toxicity, resulting in only a small fraction of compounds ultimately reaching patients 1,2. This high attrition rate highlights the limitations of traditional preclinical systems in capturing human-specific responses.

The concept of an organ-on-a-chip (OoC) emerged in the 2000s as a promising advancement in *in vitro* modeling to better emulate human physiology 3. Soon after, the concept of multi-organ-on-a-chip (MOoC) was introduced as a more complex and accurate model that includes the dynamic crosstalk and systemic circulation between different organs 4. Traditional *in vitro* methods can provide some insight into the cellular responses and local toxicities of drugs, while animal models provide further detail into the pharmacokinetics and immune or metabolic interactions of a whole organism. However, while these both serve as a baseline for preclinical evaluation, organ-on-chip and multi-organ-on-chip technologies offer models more relevant to humans with the potential to reduce cost and attrition at this early stage 5,6. Major avenues through which drugs fail in testing include poor systemic distribution, limited absorption, or unexpected organ-specific toxicity. As such, the appeal of MOoCs lies in their ability to predict these failures earlier in the drug development process and serve as a human-relevant alternative for preclinical testing and screening.

We will review recent developments in MOoC platforms, focusing on cutting-edge advances including: iPSC-derived platforms for patient-specific disease modeling, physiologically interconnected organ systems spanning neurological (gut-brain axis, neurovascular unit, neuromuscular junction), cardiovascular, hepatic, and renal compartments, advanced biosensing and real-time analytical monitoring, 3D bioprinted tissue constructs with biomimetic extracellular matrices, and scalable high-throughput screening formats. We will examine how MOoCs have been applied to study diverse drug delivery strategies, including viral vectors, nanoparticles (NPs), extracellular vesicles (EVs), cell-based therapies, and biomaterials. By evaluating representative examples across these modalities, we aim to provide a comprehensive perspective on how MOoCs contribute to overcoming delivery barriers and modeling therapeutic responses in physiologically relevant contexts. Finally, we will discuss the outlook of MOoCs as transformative platforms for future drug delivery and translational medicine, emphasizing their potential to bridge the gap between preclinical evaluation and clinical success.

To fully appreciate the capabilities of multi-organ systems, it is essential to understand the foundational principles of organ-on-a-chip technology. Single-organ systems and their underlying engineering approaches have been extensively reviewed elsewhere 3,6-11. These reviews provide comprehensive coverage of microfluidic device design, cell culture techniques, material selection, and validation methods that underpin all OoC platforms.

Key literature we discussed in this review was identified using search databases, including PubMed, Web of Science, and Google Scholar. Key search terms included a combination “multi-organ”, “multi-organ-on-a-chip”, “interconnected MPS”, “drug delivery”, “ADME”, with a focus on identifying field-defining reviews, and an emphasis on key studies published within the last 10 years.

## 2. Bioengineering multi-organ-on-a-chip platforms

While single-organ-on-a-chip systems have demonstrated remarkable capabilities in replicating organ-level functions, multi-organ integration addresses a critical limitation: the absence of intersystem crosstalk essential for predicting whole-body drug behavior and disease progression. Pharmacokinetic processes, including absorption, metabolism, distribution, and excretion, inherently span multiple tissues. Modeling these organs in isolation provides incomplete insight, whereas integrating targeted organs with metabolic and clearance pathways offers a holistic view of drug delivery and systemic exposure.

**Figure 1:**
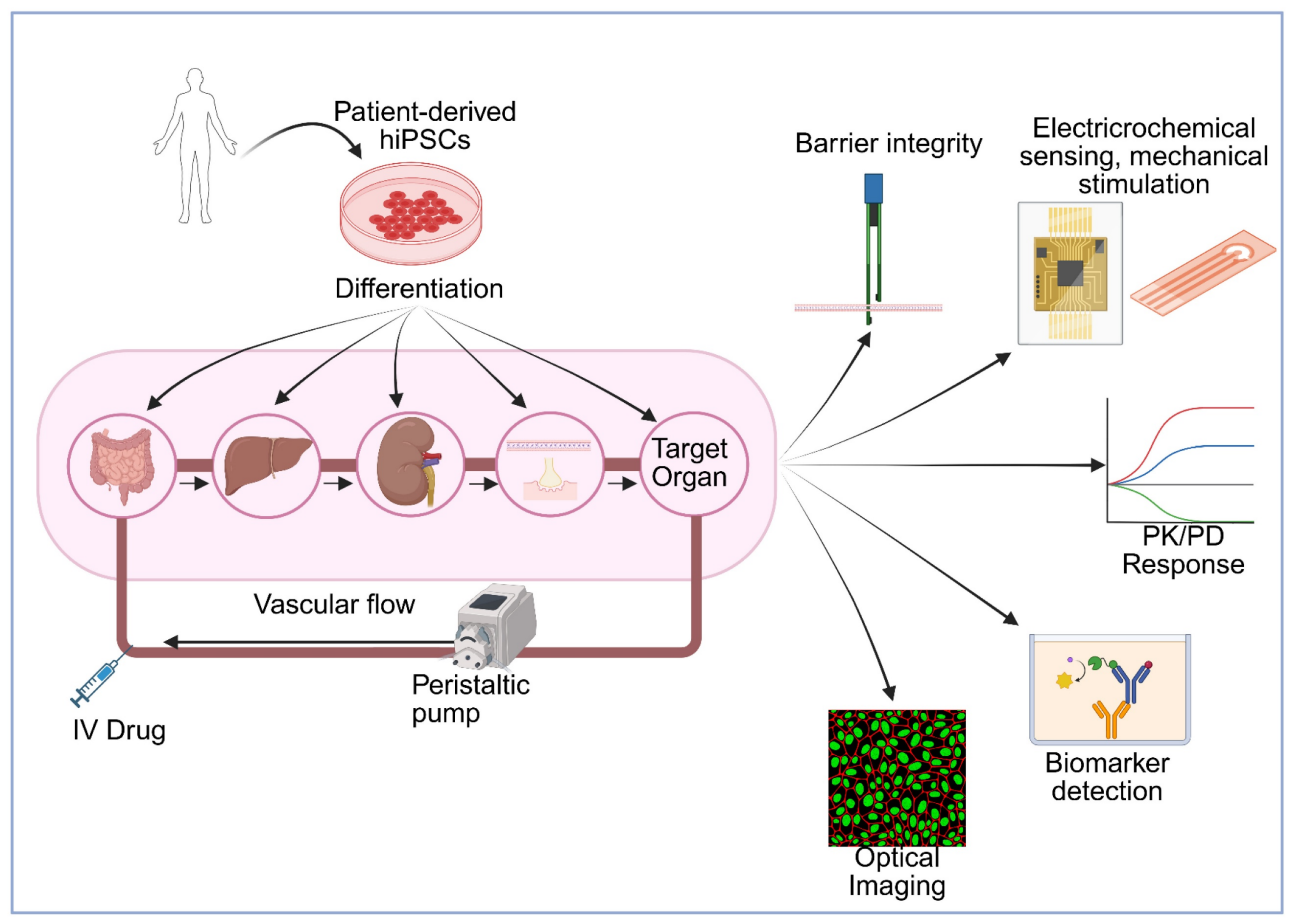
** Schematic overview of patient-specific multi-organ-on-a-chip platform opportunities for drug evaluation.** Patient-derived human induced pluripotent stem cells (hiPSCs) are differentiated into multiple organ-specific cell types and integrated into a microfluidic multi-organ system connected via vascular flow with peristaltic pump control. The platform incorporates key organs involved in drug metabolism, clearance, and barriers (gut, liver, kidney, NVU, NMJ) along with a target organ, enabling modeling of drug absorption, distribution, metabolism, and excretion (ADME). Drugs can be administered via intravenous (IV) injection or other routes. The system can integrate multiple analytical modalities including: (1) barrier integrity monitoring through transepithelial/transendothelial electrical resistance (TEER) measurements, (2) electrochemical sensing and mechanical stimulation for physiological relevance, (3) optical imaging for real-time cellular visualization, (4) biomarker detection for assessing therapeutic and toxic responses, and (5) pharmacokinetic/pharmacodynamic (PK/PD) response profiling to generate dose-response curves across different organs. This integrated platform enables prediction of human-relevant drug responses, organ-specific toxicity, and inter-organ communication under controlled physiological conditions, supporting personalized medicine approaches and reducing reliance on animal testing. Created in BioRender (https://BioRender.com/64au3m2).

The Multi-Organ-on-a-Chip, as it is often defined, is a platform of multiple tissues connected through microfluidic circulatory systems, allowing continuous perfusion, soluble factor exchange, and systemic exposure to drugs or metabolites (Figure 1). One of the first designs for a MOoC model used for absorption, distribution, metabolism, excretion (ADME) profiling included a 4-organ chip with a co-culture of intestine, liver, skin, and kidney cells 4. Through a shared recirculating medium, these organs shared a circulating fluid, mimicking blood flow. Dynamic perfusion and organ-organ communication was demonstrated for 28 days, establishing one of the first long-term, physiologically integrated human tissue platforms for drug testing. They were also capable of measuring organ-specific functionality and systemic drug metabolism, including cytochrome P activity, barrier integrity, and metabolite distribution across compartments.

Recent engineering advances have enabled the sophistication of contemporary MOoC platforms (Table 1). Progress toward physiological relevance has been achieved through synthetic hydrogels supporting 3D tissue constructs that mimic native extracellular matrix properties, with bioprinting enabling tailor-made ECMs for specific organs to enhance cell viability 12. Advances in barrier function and tissue-tissue interfaces allow for the recreation of complex native barriers such as the blood-brain barrier, incorporating tight junctions and dynamic shear stress to replicate *in vivo* permeability. Platform designs supporting long-term culture have also emerged, including pumpless circulation systems using inter-well pressure gradients 13 and closed-loop circulation that minimizes tubing and external media reservoirs to decrease media loss and contamination risk 14. Methods such as dual-flow systems enable tissue polarization, exemplified by a 96-well microfluidic platform allowing separate perfusion of apical and basolateral components in renal barrier models 15. Scaling to high-throughput formats has been achieved through plate-formatted systems, including 96-well chip arrays with pneumatic pressure control that enable independent yet synchronized operation of multiple organ units with automated liquid handling, imaging, and real-time monitoring 15,16.

Building upon these foundational multi-organ systems and engineering capabilities, contemporary MOoC platforms have evolved to address specific physiological axes and therapeutic challenges (Table 2). The following sections highlight representative systems organized by their biological focus, demonstrating how targeted multi-organ designs enable mechanistic investigation of disease pathology and drug delivery in physiologically relevant contexts.

### 2.1 Gut-brain-axis-on-a-chip

**Figure 2:**
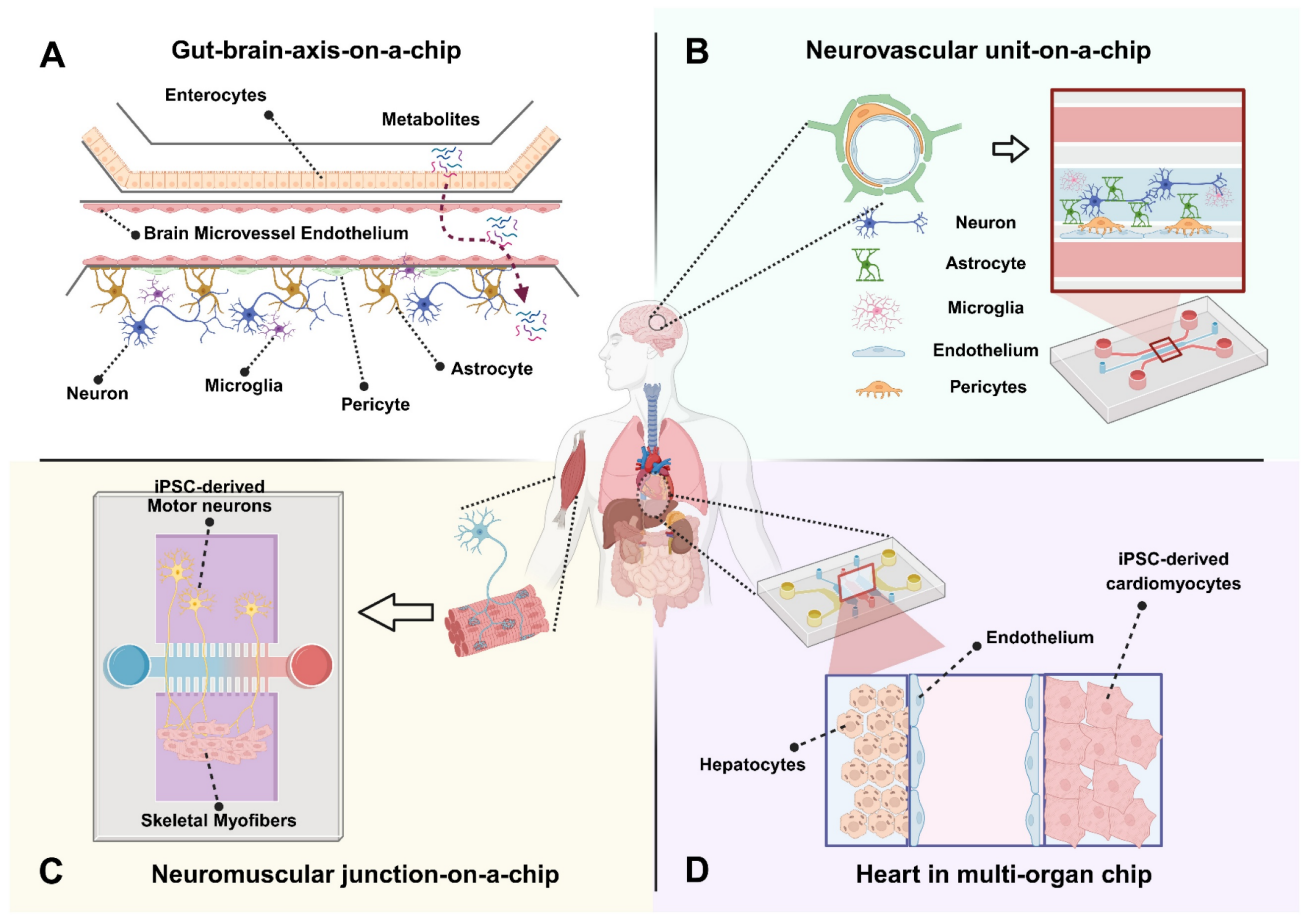
** Schematic overview of multi-organ-on-a-chip platforms for targeted drug-delivery evaluation.** A human body silhouette highlights key organs involved in systemic drug absorption, distribution, metabolism, and response. A dual-compartment microfluidic platform integrates an intestinal epithelial barrier with a BBB module. The gut chamber incorporates microvilli-bearing enterocytes that enable assessment of oral drug absorption, microbial metabolites, and epithelial transport. The downstream BBB module includes endothelial cells, astrocyte endfeet, and pericytes, enabling evaluation of molecular permeability, TEER dynamics, cytokine-mediated inflammation, and gut-derived metabolites transported to the brain (A). A perfusable endothelial microvessel is surrounded by astrocytes, neurons, microglia, and pericytes to reconstruct the structural and functional components of the human NVU. This platform allows measurement of BBB integrity, neuroinflammatory signaling, neurovascular coupling, electrophysiological activity, and drug or nanoparticle transport under controlled shear stress. The model enables patient-specific and disease-relevant investigation of ischemia, neurodegeneration, and barrier dysfunction (B). A compartmentalized microfluidic device links iPSC-derived motor neurons to aligned skeletal myofibers through directed axonal projection. This system facilitates real-time assessment of neuromuscular synaptogenesis, neurotransmission, calcium dynamics, optogenetically induced contraction, and synaptic deficits associated with neuromuscular disorders such as ALS. Functional readouts include MEA-based electrophysiology, force generation profiles, and acetylcholine receptor clustering (C). A coupled hepatocyte-cardiac platform models drug metabolism and metabolism-dependent cardiotoxicity. The liver compartment supports phase I/II metabolic processing, while the connected cardiac module contains beating cardiomyocytes with electrophysiological readouts. This integrated system enables evaluation of parent compounds, hepatic metabolites, biomarker release (BNP, troponin), and PK/PD responses under dynamic perfusion (D). Created in BioRender (https://BioRender.com/0gptvri).

The gut-brain axis is a system that has gained a lot of attention in recent years due to its relevance in neurodegeneration and inflammatory diseases. This system is particularly relevant for understanding drug delivery that utilizes an oral delivery method, as the gut is often the first barrier for absorption. In addition, with the brain having one of the most selective barriers, this model provides a substantial perspective on a full absorption to target pathway. Most gut-brain chip designs utilize modular compartments separated by permeable membranes. An important component of these designs is the creation of distinct microenvironments tailored to each organ, meeting specific needs of oxygenation, media, and flow dynamics (Figure 2A). These systems utilize distinct compartments that allow for co-culture of gut and brain cell types (typically Caco-2 or induced pluripotent stem cell (iPSC)-derived enterocytes for the gut; hBMECs, astrocytes, and pericytes for the BBB) and allow for maintenance of physiological flow rates and shear stress for both compartments 17,18.

The use of iPSC-derived cells has become of particular interest because they allow for patient-specific modeling and the generation of mature neuronal or gut epithelial subtypes, which improves disease relevance and expands the potential for personalized medicine. However, these studies also highlight limitations including the challenges of maintaining functional maturation and long-term viability in co-culture systems, as well as variability between iPSC lines.

Important analytical readouts include confirming barrier integrity for both the gut and brain compartments, typically measured using TEER and permeability assays. Additionally, in the context of inflammation, cytokine release panels (e.g., IL-6, TNF-α) can track immune responses to microbial or drug stimuli across the interface. Metabolite and neurotransmitter quantification can elucidate chemical communication across the axis, including serotonin, short-chain fatty acids, or drug metabolites. Also, live imaging and reporter assays can provide important information on the status of tight junctions, oxidative stress, or inflammatory signaling within each tissue compartment.

Few reliable gut-brain multi-organ platforms have been made at this point, as the majority of brain-related OoC studies have been focused on creating an accurate model of the blood-brain barrier. Some current gut-brain chip designs attempt to link gut models with BBB models and include confirmation of formation of both barriers and transport observations of known microbial products and gut-derived exosomes 19. Additionally, gut-liver-brain models have been examined in the context of Parkinson's disease, as short-chain fatty acids are thought to induce changes in neurodegenerative pathology. These studies suggest systemic metabolite transfer and immune activation may influence CNS phenotypes. Additionally, a gut-brain-immune axis model has been proposed to better capture bidirectional signaling involving circulating immune cells, though few models have successfully integrated all three components in dynamic co-culture 17.

In a recent example of functional multi-organ modeling, a gut-brain chip was constructed and challenged with donepezil, an Alzheimer's medication 20. In this study, they demonstrated the ability to monitor gut and brain barriers in real time using permeability assays and integrated biosensors, and they were able to monitor drug absorption from the gut to the brain under dynamic perfusion conditions. They also observed pharmacodynamic effects including acetylcholine modulation and downstream neuroinflammatory markers.

Despite these advances, there remain significant limitations with current models. As mentioned, current work on brain-on-a-chip units is still mostly focused on simulating a complete and accurate blood-brain barrier, which is a necessary advancement in the path of producing a fully functional neurovascular unit (NVU) before integrating into a multi-organ model. Another roadblock is establishing a complete gut microbiome, mostly due to the difficulties in establishing a unique anaerobic coculture for the gut microbiome while also allowing for aerobic brain tissue viability and media recirculation. Furthermore, iPSC-based approaches offer promise for disease models and personalized testing, but there are still challenges with cost and creating reproducible cell populations and barrier properties 21. In terms of future directions, efforts are focused on integrating immune cells, simulating microbiomes, and developing hormonal cues to better reflect the bidirectional complexity of the gut-brain axis.

### 2.2 Neurovascular unit (NVU)-on-a-chip

The neurovascular unit (NVU) represents the fundamental structural and functional interface linking the cerebral vasculature with neural and glial networks 22,23. NVU-on-a-chip platforms recapitulate this multicellular microenvironment by integrating brain microvascular endothelial cells, pericytes, astrocytes, neurons, oligodendrocytes and often microglia within microfluidic architectures (Figure 2B). These platforms enable simulation of complex *in vivo* cellular interactions that govern blood-brain barrier (BBB) integrity, cerebral metabolic processes, and neuroimmune interactions 24. Unlike conventional BBB-on-chip models that primarily focus on endothelial barrier properties, NVU chips incorporate neuronal and glial components to reproduce bidirectional signaling between vascular and parenchymal compartments, thereby bridging vascular physiology with neural function and pathology 25,26.

Recent engineering advances have enabled three-dimensional (3D) co-culture configurations in which perfusable endothelial tubes enveloped by astrocytes and neurons embedded within extracellular matrix hydrogels, allowing astrocytic endfeet to contact the vascular wall and neuronal processes to extend through the matrix, facilitating spontaneous synaptic activity and physiologically relevant signaling 27,28. Controlled microfluidic perfusion generates shear stress that promotes endothelial tight-junction assembly and transporter expression, thus improving barrier fidelity and enabling quantitative assessment of molecular permeability, neuroinflammation, and neurovascular coupling under defined hemodynamic conditions 29-31.

NVU-on-a-chip models have been leveraged to study a range of central-nervous-system (CNS) disorders including ischemic stroke, Alzheimer's disease (AD), and amyotrophic lateral sclerosis (ALS). Transient ischemia can be reproduced by regulating flow or oxygen tension, allowing evaluation of barrier breakdown, excitotoxicity, and neurorestorative interventions such as stem-cell-based repair 32. iPSC-derived human NVU chips carrying familial AD mutations exhibit hallmark phenotypes such as β-amyloid accumulation, pericyte dysfunction, and impaired barrier integrity, mirroring patient-specific pathophysiology 33-35. These personalized microphysiological systems enable mechanistic dissection of endothelial-glial signaling and preclinical screening of BBB-penetrant therapeutics, neuroprotective compounds, and nanoparticle-based delivery vehicles 36-38.

Integration of biosensing and real-time analytics further enhances the translational potential of NVU chips 39-41. Embedded microelectrodes and impedance sensors permit continuous monitoring of trans-endothelial electrical resistance (TEER), neuronal firing, and cytokine dynamics, facilitating kinetic profiling of drug transport and immune-vascular interactions in defined microenvironments 42-44. Exposure to inflammatory cytokines or amyloid-β oligomers induces quantifiable barrier leakage and endothelial toxicity, reproducing early pathological events of neurodegenerative disease progression 45.

Despite these advances, achieving standardized, long-term NVU-on-a-chip systems remains a major challenge. Maintaining co-culture stability, incorporating microglia without excessive inflammatory drift, and establishing reproducible manufacturing pipelines are ongoing priorities. Future designs are expected to integrate metabolic coupling with peripheral organ modules, such as gut or liver, to capture systemic influences on the brain and expand applicability for CNS drug-delivery testing 21,46. Furthermore, incorporating glymphatic-like fluid dynamics to replicate cerebrospinal-interstitial exchange and metabolic waste clearance, together with the use of patient-derived iPSCs to reflect interindividual variability in neurovascular function, represents a crucial direction for future translational research 47-49.

### 2.3 Neuromuscular junction (NMJ)-on-a-chip

Neuromuscular junction (NMJ)-on-a-chip systems emulate the synaptic interface between motor neurons and skeletal muscle fibers, creating a microphysiologically relevant environment for studying signal transmission, excitation-contraction coupling, and neuromuscular disorders. Microfluidic compartmentalization allows directed axonal projection from neuronal chambers toward muscle compartments, enabling compartment-specific control of chemical gradients and trophic cues (Figure 2C) 50,51. The integration of 3D hydrogel matrices and aligned myofiber scaffolds contributes to structural fidelity, physiological contractility, and mechanical feedback 52,53. These engineered systems combined with microelectrode arrays (MEAs) or optogenetic stimulation tools provide precise quantitative readouts of neurotransmission, muscle contractility, and synaptic function under defined perfusion conditions 54,55.

The application of iPSC technology has advanced NMJ modeling by enabling patient-specific neuromuscular disease reconstruction 56-58. Recently, rapid iPSC-based NMJ-on-a-chip system was introduced wherein cryopreserved human motor neurons and skeletal myotubes formed mature synaptic junctions within 12 days, recapitulating SOD1 mutation-related degeneration observed in amyotrophic lateral sclerosis (ALS) 59. This system demonstrated functional NMJ coupling validated through α-bungarotoxin labeling and electrophysiological recordings. Similarly, Osaki et al presented an ALS-on-a-chip integrating iPSC-derived motor neurons with optogenetically controlled synaptic inputs to model glutamate excitotoxic injury and its impact on NMJ integrity, thereby linking neurodegeneration to synaptic dysfunction 60. More recently, geometrically engineered assembloid-on-a-chip systems have expanded this framework by incorporating three-dimensional, self-organizing co-cultures of motor neurons, Schwann cells, and muscle fibers, achieving synchronized optogenetically evoked contractions that mimic *in vivo* neuromuscular circuitry 61. Comparative analyses across these models highlight that while dense neuronal populations enhance motor neuron viability, optimal NMJ formation often relies on lower neuronal density to promote selective neuron-to-muscle connectivity.

NMJ-on-a-chip systems employ diverse analytical modalities to quantify pre- and postsynaptic functionality. MEA-based recordings provide electrophysiological insights into presynaptic burst patterns and muscle depolarization, whereas calcium imaging enables real-time visualization of excitation-contraction coupling kinetics 62,63. Molecular assays, such as acetylcholine receptor clustering, agrin and MuSK signaling assessment, and postsynaptic membrane stability tests, serve as biomarkers of synaptic maturation and responsiveness 64. Beyond disease modeling, emerging bioactuated applications integrate NMJ tissues into soft robotic and neuromorphic frameworks, enabling biologically controlled mechanical outputs 65. The integration of machine learning algorithms and biosensor feedback loops further refines automatic detection of synaptic fatigue, transmission failure, and plasticity, positioning NMJ-on-chip models as valuable testbeds for neurotoxicology and regenerative drug discovery 66,67.

Although substantial progress has been made, several challenges remain in sustaining long-term synaptic integrity, coordinated cell survival, and functional adaptability during prolonged stimulation. Future directions involve incorporating myelinating Schwann cells and fibroblasts to restore trophic support and synaptic repair pathways, as well as coupling NMJ units with vascular or hepatic modules in multi-organ circuits to model metabolic and inflammatory influences on neuromuscular health 68,69. Simultaneously, efforts are ongoing to enhance throughput via automated optical stimulation and AI-assisted image analysis, enabling large-scale screening of therapeutic compounds targeting neuromuscular transmission pathways.

### 2.4 Heart in multi-organ platforms

The heart plays a central role in multi-organ physiology and pharmacokinetics, acting as both a target and mediator of systemic drug circulation. Integrating a heart-on-a-chip (HoC) into MOoC systems is particularly critical for evaluating drug efficacy and cardiotoxicity under physiologically relevant flow and mechanical conditions 70,71. HoC devices replicate the myocardium's electrophysiological and contractile properties using microfluidic and tissue-engineering approaches, which enable real-time evaluation of cardiac function, rhythm, and metabolic coupling under controlled perfusion 72,73. By embedding cardiomyocytes, often derived from iPSCs, within mechanically active microenvironments, these systems recapitulate the beating and electromechanical synchronization characteristic of native cardiac tissue, surpassing traditional 2D culture limitations (Figure 2D) 74,75.

In multi-organ contexts, the HoC often interfaces with key metabolic and regulatory organs such as the liver, kidney, or brain to evaluate systemic pharmacokinetic behavior and multi-organ drug responses 76,77. For example, heart-liver chip models have been successfully used to assess cardiotoxic effects of hepatic drug metabolites. Co-cultured liver-heart chip could simulate clomipramine-induced cardiotoxicity dependent on hepatic metabolic processing, thereby illustrating the predictive power of inter-organ coupling. Such systems allow evaluation of both primary cardiac drug effects and secondary, metabolism-driven toxicity, which are otherwise challenging to predict using single-organ assays 78,79.

More advanced integrations include neuro-cardiac-on-a-chip constructs, which investigate bidirectional signaling between neurons and cardiomyocytes 80,81. These hybrid systems recreate neuro-cardiac junctions essential for understanding arrhythmias and stress-induced heart dysfunctions 82,83. The combination of human iPSC-derived cardiomyocytes and neurons within microfluidic co-culture platforms has enabled dynamic, patient-specific modeling of autonomic regulation in cardiac pathophysiology 84,85.

**Figure 3:**
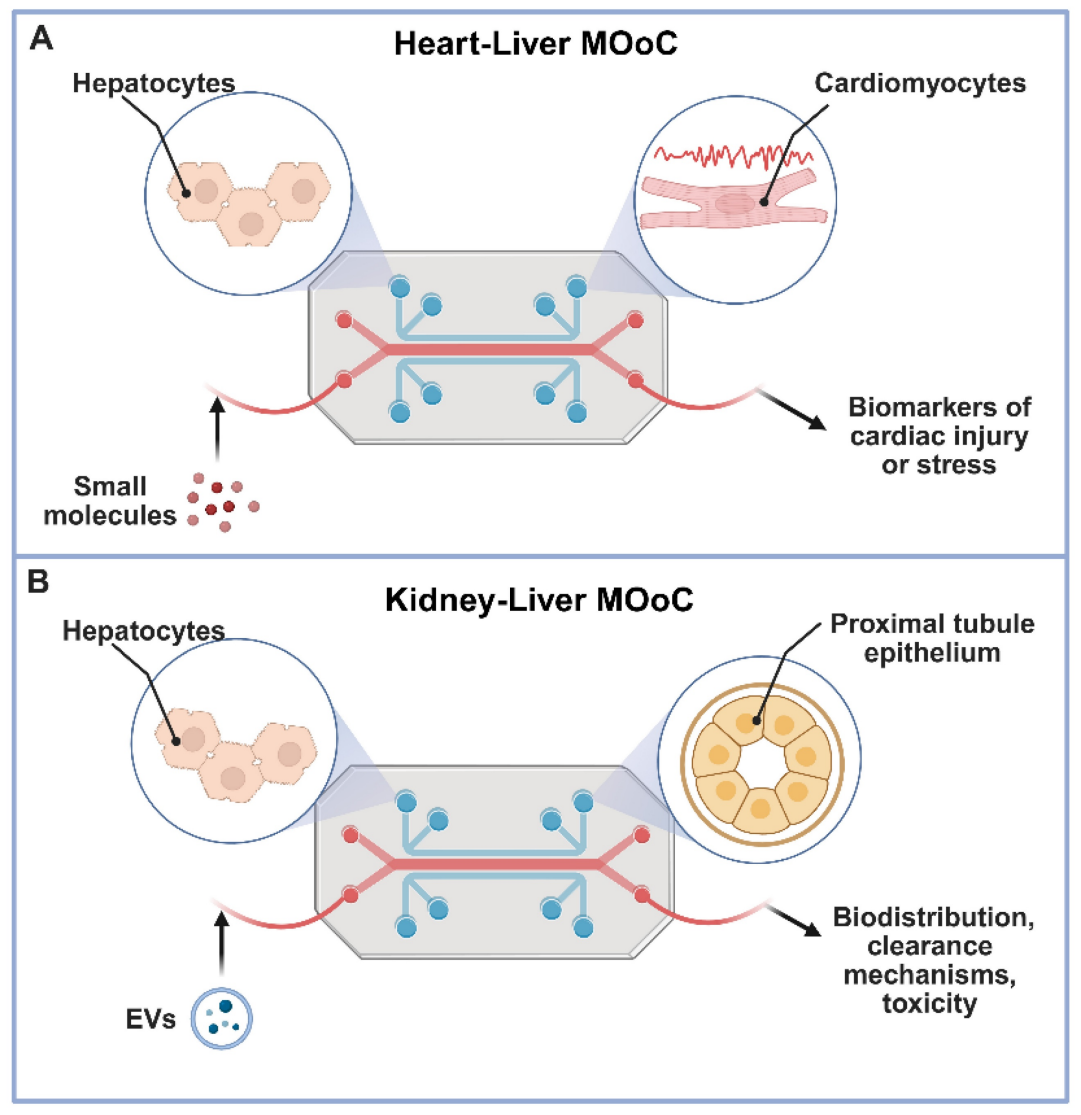
** Multi-organ-on-a-chip systems for comprehensive drug delivery evaluation.** This figure illustrates the power of multi-organ-on-a-chip (MOoC) platforms in evaluating advanced drug delivery strategies by modeling inter-organ communication and systemic effects under physiologically relevant conditions. Panel A depicts a Heart-Liver MOoC, demonstrating its utility in assessing metabolism-driven cardiotoxicity, where a parent drug is metabolized in the liver, and the resulting metabolites circulate to induce potential adverse effects in the heart 92. Panel B showcases a Kidney-Liver MOoC, highlighting its role in tracking the biodistribution and renal impact of agents like extracellular vesicles (EVs) or natural compounds, which are processed by the liver before circulating to the kidney, allowing for the evaluation of clearance, nephrotoxicity, or therapeutic outcomes 98. Together, these examples underscore how MOoC systems provide crucial, human-relevant insights into the complex systemic fate and organ-specific responses to diverse advanced therapeutics. Created in BioRender (https://biorender.com/qe5ibkt).

Analytical endpoints in heart-integrated MOoC setups combine electrical, biochemical, and mechanical measurements. Typical readouts include contractile amplitude and frequency, field potential duration, and calcium transients to assess tissue excitability and drug response 86-88. Simultaneous measurement of biomarkers such as troponin I or brain natriuretic peptide (BNP) provides indicators of cardiac injury or stress 89,90. Integration of biosensors directly within these systems enables real-time monitoring of beating patterns and metabolic fluxes under cyclical perfusion. Additionally, liver-heart coupled systems have adopted metabolomics and electrophysiological metrics to distinguish between reversible and irreversible toxicity profiles, improving preclinical evaluation accuracy 9,91. Multi-organ circuits containing hepatic, cardiac, and vascular modules have also been developed to replicate systemic circulation and realistic drug distribution profiles, moving closer to whole-body pharmacokinetic simulation (Figure 3A) 92,93.

### 2.5 Liver in multi-organ platforms

The liver is extremely relevant in drug metabolism, specifically with Phase I and II reactions. It is responsible for the biotransformation of a wide range of compounds, including oxidation, reduction, conjugation, and hydrolysis reactions that can either activate or inactivate therapeutic agents. Drugs taken orally, after absorption through the intestine, encounter the “first-pass” effect when passing through the liver, where a large portion of the drug is metabolized and removed from the bloodstream. Since this organ can have significant impact on the bioavailability of a drug, it is crucial to understand how hepatic metabolism alters the active form, concentration, and half-life of administered compounds, and it demonstrates the need to link this system to upstream (such as gut and skin) and downstream (such as kidney and tumor) modules in order to accurately predict metabolic effects.

We return to the early four-organ-chip model to understand how the inclusion of liver contributes to systemic drug processing. This system linked intestine, liver, kidney, and skin tissues via dynamic flow, and demonstrated metabolic activity sustained for over four weeks. This liver model showed some enzymatic activity and albumin production comparable to *in vitro* monocultures and demonstrates that some liver functionality is capable in liver-on-chip models 4.

Experiments that explore the liver-on-chip module as a metabolic gatekeeper observed controlled changes in concentration of parent drugs and metabolites at downstream heart, tumor, and lung modules, in the context of chemotherapeutics and targeted small molecules 94,95. These studies demonstrated that hepatic metabolism could either reduce drug efficacy at the tumor site or amplify off-target toxicity at cardiac tissues, depending on the metabolic fate of the compound. The lung-liver platform additionally shows that cytokines produced in one tissue would affect barrier integrity and response in the other, indicating inflammatory crosstalk between organ modules in response to metabolic products or stress 94.

A key consideration in this system is the need for long-term maintenance of hepatic function and viability under continuous flow. Since chronic exposure is relevant in many toxicological and pharmacokinetic studies, it is necessary to ensure that these systems can maintain function for extended time periods. Use of dynamic perfusion systems such as peristaltic micropumps or pumpless gravity-driven flow setups can assist with the required viability and function. In terms of future directions, there is growing relevance in integrating iPSC-derived hepatocytes, modeling disease-specific liver phenotypes, and mimicking spatial heterogeneity in liver function 6.

### 2.6 Kidney in multi-organ platforms

The kidney plays an essential role in eliminating hydrophilic drugs from the bloodstream, and as such can have a significant effect on systemic clearance and drug half-life. Drug-relevant processes that occur within the kidney, specifically in the nephrons, include glomerular filtration and phase III transporter-mediated secretion and reabsorption, all processes which can influence drug efficacy and toxicity.

One of the key components of getting accurate pharmacokinetic information from a kidney model is the presence and detection of key transporter activity. An accurate kidney model requires polarized proximal tubule epithelium expressing active transporters such as OATs, OCTs, and MATE family transporter proteins 96. In the work done by Chang et al., they demonstrate that maintaining dynamic fluid flow is necessary in this system to simulate shear stress and produce functional differentiation, resulting in transporter expression. They further emphasize that fluidic shear is essential for sustaining this expression over time and preventing de-differentiation of epithelial cells 97.

In an example of multi-organ interaction, Nguyen et al. showed a functional kidney-liver on-a-chip model altering the biodistribution of extracellular vesicles and natural compounds such as silymarin and berberine. They demonstrated crosstalk between the liver and kidney model and the effect of liver dysfunction on the kidney model, which mirrored clinically relevant hepatorenal effects observed in diseases like MAFLD. They also exposed the system to natural compounds, which were metabolized by the liver and then received by the kidney, allowing them to assess nephrotoxicity, clearance, and the downstream impact of liver-derived metabolites on renal function (Figure 3B) 98.

Many current MOoC designs rely on computational or other artificial modes of elimination that may serve as a rudimentary stand-in for renal clearance but lack physiologically relevant transporter expression and inter-organ feedback. Future directions should integrate functional kidney modules with glomerular and tubular components, real-time readouts of renal transport, and disease-relevant models that can better emulate impaired renal clearance.

### 2.7 Organoids in multi-organ platforms

Three-dimensional organoids have emerged as powerful *in vitro* models that recapitulate native tissue cytoarchitecture, multicellular organization, and developmental patterning in ways that monolayer cultures or simplified co-culture systems cannot achieve. Derived from human iPSCs or primary patient biopsies, organoids self-organize into physiologically relevant structures that recapitulate spatial gradients, stem cell niches, and functionally distinct cellular subtypes 99. While traditional organoid systems lack vascular perfusion and often exhibit diffusion-limited nutrient and oxygen transport, integrating organoids within microfluidic platforms has enabled a new class of organoid-on-a-chip technologies that merge the physiological strengths of 3D tissues with the controlled perfusion and barrier dynamics of microfluidics 100.

Microengineered organoid-on-chip platforms have been reported for a range of tissues, including gut, liver, kidney, brain, and tumor microenvironments, each offering enhancements over 2D or planar engineered constructs 100. For example, the incorporation of primary or iPSC-derived intestinal organoids into microfluidic channels has been shown to promote crypt-villus architecture, mucus layer formation, and regionalized epithelial maturation under physiologically relevant flow and shear stress 101,102. Similarly, cerebral and neurovascular organoid-on-chip systems support increased neuronal maturation, functional network activity, and improved modeling of neurodevelopmental or neurodegenerative processes by combining organoid self-organization with perfusable vascular-like structures 103-105. These constructs represent important complements to the BBB- and NVU-on-chip technologies described earlier and serve as promising tools for investigating neuronal-vascular coupling, inflammatory signaling, and patient-specific neurological phenotypes.

Organoid models also provide substantial advantages for metabolic and clearance organs, where 3D architecture, ECM-rich microenvironments, and zonated functional domains are key drivers of physiological function. Multi-organ systems incorporating liver or kidney organoids have demonstrated improved long-term viability and metabolic fidelity compared to 2D or scaffold-seeded tissues. A notable example includes a kidney-liver organoid-based MOoC used to study extracellular vesicle biodistribution and hepatorenal crosstalk, revealing disease-relevant alterations in clearance and toxicity pathways under dynamic perfusion 98. Tumor organoid-on-chip systems have similarly advanced the study of cancer drug responses by enabling vascularized, stromalized 3D tumor architecture with improved prediction of drug penetration, therapeutic efficacy, and off-target toxicity 106-109; capabilities that are difficult to achieve in static organoid cultures alone.

The integration of organoids into multi-organ microphysiological circuits represents an important step toward achieving physiologically scalable multi-tissue interactions. Organoid-on-chip models have been applied to study gut-brain axis communication, NVU dysfunction, metabolic disease, and tumor-immune dynamics, offering enhanced biological fidelity for drug absorption, metabolism, and toxicity evaluation. Their patient-specific nature also positions them as valuable platforms for personalized medicine and precision drug screening, particularly for modalities such as biologics, nanoparticles, gene therapies, and extracellular vesicles that interact with complex 3D tissue structures and restrictive biological barriers.

Despite these advantages, several challenges remain. Organoids exhibit batch-to-batch variability, and their self-organized geometry can complicate standardization and reproducibility. Achieving robust vascular integration, scaling organoid size for perfusion compatibility, and incorporating immune or stromal elements remain active areas of research. Nevertheless, ongoing engineering advances, including organoid vascularization strategies, guided morphogenesis, and controlled spatial seeding within microfluidic compartments, continue to improve consistency and functional maturation. As these techniques are refined, organoid-on-a-chip platforms are expected to play a central role in next-generation multi-organ systems by bridging the gap between developmental biology, engineered microenvironments, and human-relevant drug evaluation.

### 2.8 Pharmacokinetic validation and computational approaches in multi-organ platforms

MOoC platforms are increasingly positioned as human-relevant tools for improving prediction of ADME. However, their translational utility critically depends on the extent to which chip-derived data can quantitatively reproduce PK parameters observed *in vivo* or in clinical settings. In recent years, the field has progressed beyond qualitative demonstrations toward more rigorous, quantitative validation through integration with physiologically based pharmacokinetic (PBPK) modeling frameworks 110,111.

A landmark study by Herland et al. demonstrated that fluidically coupled, vascularized human organ chips could quantitatively predict human plasma concentration-time profiles for multiple drugs 112. By linking liver, intestine, kidney, and other organ modules under shared circulation and coupling experimentally measured transport and metabolism parameters with PBPK models, the authors achieved close agreement with clinical PK data across different compounds. This work provided one of the first demonstrations that MOoC-derived parameters, such as intrinsic clearance, permeability, and organ-specific extraction, can be directly translated into whole-body PK predictions.

Subsequent studies have further clarified the role of MOoC platforms as experimental parameter generators for PBPK modeling rather than standalone predictors 113,114. MOoC systems can provide human-specific inputs including metabolic turnover rates, barrier permeability coefficients, transporter activity, and time-resolved concentration profiles. When incorporated into PBPK frameworks, these parameters improve mechanistic interpretability and reduce uncertainty associated with interspecies scaling, particularly for drugs with complex metabolism or barrier-limited distribution 115.

Despite these advances, important limitations remain. Quantitative concordance is strongest for small-molecule therapeutics, while validation for biologics, nanoparticles, and gene-based modalities is still emerging. Challenges related to physiological scaling of organ size and flow, material-dependent drug adsorption, simplified renal and immune clearance pathways, and limited culture duration continue to constrain absolute PK accuracy. As emphasized by Ingber 116, regulatory agencies currently view organ-chip technologies not as replacements for animal models, but as complementary, decision-support tools that can strengthen mechanistic confidence and inform translational decision-making when used within defined contexts.

In recent years, computational approaches have progressed beyond conceptual demonstrations and are now being practically coupled with MOoC-derived data to inform physiologically based pharmacokinetic (PBPK) models. For example, human liver-on-chip systems have been used to generate intrinsic clearance and metabolic turnover rates that accurately parameterize PBPK models, yielding plasma concentration-time profiles concordant with clinical observations across multiple small-molecule drugs 115,117. Similarly, intestine-liver two-organ chips have provided experimentally derived permeability, efflux ratios, and first-pass metabolism constants that, when incorporated into mechanistic absorption models, improve the prediction of oral bioavailability and hepatic extraction 117,118. BBB-on-chip platforms have generated human-relevant drug permeability coefficients and TEER values that integrate directly into CNS PBPK frameworks, enabling quantitative prediction of brain partitioning and exposure. Chip-based hepatic models have also produced transporter and enzyme inhibition constants suitable for drug-drug interaction modeling, with PBPK simulations accurately reproducing known clinical interactions 115. Together, these examples highlight that MOoC platforms can now supply validated, human-specific parameters for computational models, including clearance, permeability, metabolism, partitioning, and transport kinetics. Despite this progress, several limitations remain. Current efforts often rely on simplified chip geometries, incomplete representation of systemic clearance pathways, or short culture durations that limit long-term PK/PD prediction 118, and scaling non-physiological flow rates, surface-area-to-volume ratios, and organoid sizes into whole-body PBPK models remains challenging 115. Moreover, integration of large biologics, nanoparticles, gene therapies, and cell-based modalities into PBPK frameworks is only beginning to emerge, given the limited availability of quantitative chip-derived ADME data. Continued development of computational models informed by rigorously standardized MOoC parameters will be essential for enabling predictive human-relevant PK/PD estimations in the future.

## 3. Microphysiological systems-driven advances in drug delivery strategies

In this section, we focus on how microphysiological systems (MPS), which are engineered *in vitro* platforms that recapitulate key structural, mechanical and functional features of human tissues using microfluidics and advanced biomaterials, can be leveraged to evaluate next generation drug delivery strategies. Within this broad category, MOoC platforms represent a subset of MPS in which multiple organ tissue or organ modules are interconnected by a shared perfusion circuit to model pharmacokinetics and inter-organ crosstalk. Unless otherwise noted we use “MPS” to refer to both single-organ and multi-organ platforms, and “MOoC” specifically for interconnected multi-organ systems.

### 3.1 Stem cell-based delivery in MPS

Stem cells have emerged as promising therapeutic carriers owing to their intrinsic regenerative capacity, tissue-homing properties, and immunomodulatory potential 119. Initially, many strategies focused on using whole cells as delivery vehicles engineered to secrete therapeutic proteins, cytokines, and growth factors at disease sites 120. However, the intrinsic complexity and heterogeneity of live cells pose substantial challenges for manufacturing, quality control and long-term safety. As a result, the primary use of stem cells in drug delivery applications shifted towards harnessing stem-cell derived extracellular vesicles (EVs), which we review in Section 3.4. Regardless, here we briefly describe whole-cell drug delivery approaches in the literature and highlight potential MPS applications.

Neural stem cells (NSCs), for example, demonstrate translational relevance for central nervous system (CNS) disorders, where they can migrate across the blood-brain barrier (BBB) and deliver therapeutic payloads to sites of pathology 121. Mesenchymal stem cells (MSCs) have also been studied as delivery vehicles in oncology due to their ability to home to tumors 122. Taking advantage of this feature, researchers have investigated genetically modified MSCs as targeted delivery systems, engineering them to secrete therapeutic proteins such as interferon (IFN)-β or TRAIL within the tumor microenvironment to suppress tumor growth 122,123. In another example, polymer-based gene delivery systems, such as those using poly(beta-amino ester) (PBAE) nanoparticles, can be used to *ex vivo* non-virally engineer MSCs to secrete VEGF to induce angiogenesis and tissue regeneration 124 or secrete BMP4 to target and turn off brain tumor initiating cells, putative brain cancer stem cells, to extend brain cancer survival in mice 123.

Nevertheless, critical hurdles remain beyond manufacturing, including immune rejection, uncontrolled differentiation, and tumorigenic risk. These limitations have motivated a shift toward acellular stem cell-derived products, particularly EVs, but also highlight the need for human-relevant platforms to systematically evaluate whole-stem cell therapies where they remain of interest. Indeed, MPS and MOoC platforms offer controlled microenvironments in which stem cell-based therapies can be evaluated for homing, engraftment, and functional activity, while simultaneously providing an opportunity to assess biodistribution, safety, and therapeutic efficacy across interconnected organ models under physiologically relevant conditions 125.

### 3.2 Gene and nucleic acid delivery in MPS

Gene delivery technologies have rapidly progressed through viral and non-viral approaches. Viral vectors such as adeno-associated viruses (AAVs) and lentiviruses remain the most widely employed owing to their efficiency and tissue tropism, with next-generation capsids engineered to minimize immunogenicity and enhance payload specificity 126. Non-viral carriers, including lipid nanoparticles (LNPs) and polymeric nanoparticles (PNPs), as well as physical methods of delivery, such as electroporation, are increasingly being investigated due to their advantages for safety, manufacturing scalability with a low cost of goods, large cargo capacity, flexible targeting capability, and tunable gene expression durability 121,127. More recently, CRISPR/Cas9 and other precision editing platforms have been incorporated into delivery systems, improving on-target activity for durable changes in gene expression while reducing off-target events 128.

For instance, an iPSC-derived retina-on-a-chip system was recently used to characterize AAVs based on their transduction efficacy and cell tropism 129. While many studies have interrogated AAV-driven gene delivery in human organoid models, this study was the first to use MPS to test AAV transduction efficacy (Table 3) 129,130. This work illustrates the great potential of MPS technologies as gene delivery screening platforms, allowing for the evaluation of vector biodistribution, organ-specific uptake, and immunological responses, which are factors that are often insufficiently captured in static culture or animal models 131.

Indeed, MPS/MOoC platforms could provide a marked advantage for quantifying the transduction efficiency, cell-type specificity, and durability of expression of gene and nucleic acid therapeutics in human-relevant tissues under controlled flow and barrier conditions 132. When extended to MOoC configurations that link metabolic and clearance organs with target tissues, these systems can reveal patterns of off-target transduction in a single experiment, helping to de-risk gene delivery vectors and refine dosing strategies before clinical studies.

### 3.3 Nanoparticle and nanocarrier delivery in MPS

Nanoparticles (NP) are among the most versatile and widely investigated drug delivery systems. Polymeric, lipid-based, metallic, silica, and hybrid nanoparticles have been engineered to encapsulate diverse therapeutic cargos, regulate release kinetics, and prolong circulation 133. Surface functionalization with antibodies, peptides, or aptamers enables active targeting, while stimuli-responsive designs provide spatiotemporal control over cargo release 134. Despite these advances, challenges such as immune clearance and systemic instability persist. Biomimetic nanoparticles, such as red blood cell membrane-coated systems, are being developed to circumvent immune recognition 135. Biomimetic particles can mimic particle size, shape, and surfaces of naturally occurring biological cells or particulates to have improved biodistribution and targeting properties 136.

MPS technologies offer advanced platforms for preclinical assessment of engineered nanoparticles by mimicking the complex structure-function of human organs, and by investigating how properties like size, shape and surface charge affect their targeted delivery and therapeutic efficacy (Table 3) 133,137. Studies have used tumor-on-a-chip models to show that smaller nanoparticles penetrate tumor tissue more effectively 138. Moreover, the ability to replicate the microenvironment, such as fluid shear stress and 3D tissue architecture allows for more accurate prediction of how nanoparticles will behave *in vivo* compared to 2D cultures 139. MPS models are key for evaluating the transport properties of new medicines, including when the medicines have unique sustained release profiles or unique extracellular interactions. For example, vascularized tumor-on-a-chip systems can best quantify drug flux through stroma and tumor in a way that is predictive of patient responses, especially if patient-specific cells are utilized 140. Beyond efficacy, MPS models have been used to test potential nanoparticle toxicity and safety. For example, lung-on-a-chip systems demonstrated that mechanical forces from breathing can worsen the adverse effects of silica nanoparticles, which are toxic to the alveolar-capillary barrier 141. Indeed, MPS platforms enable detailed assessment of NP biodistribution, cellular uptake, clearance pathways, and toxicity under physiologically relevant fluid dynamics, thereby improving translational accuracy compared to traditional *in vitro* or *in vivo* testing 142,143.

**Figure 4:**
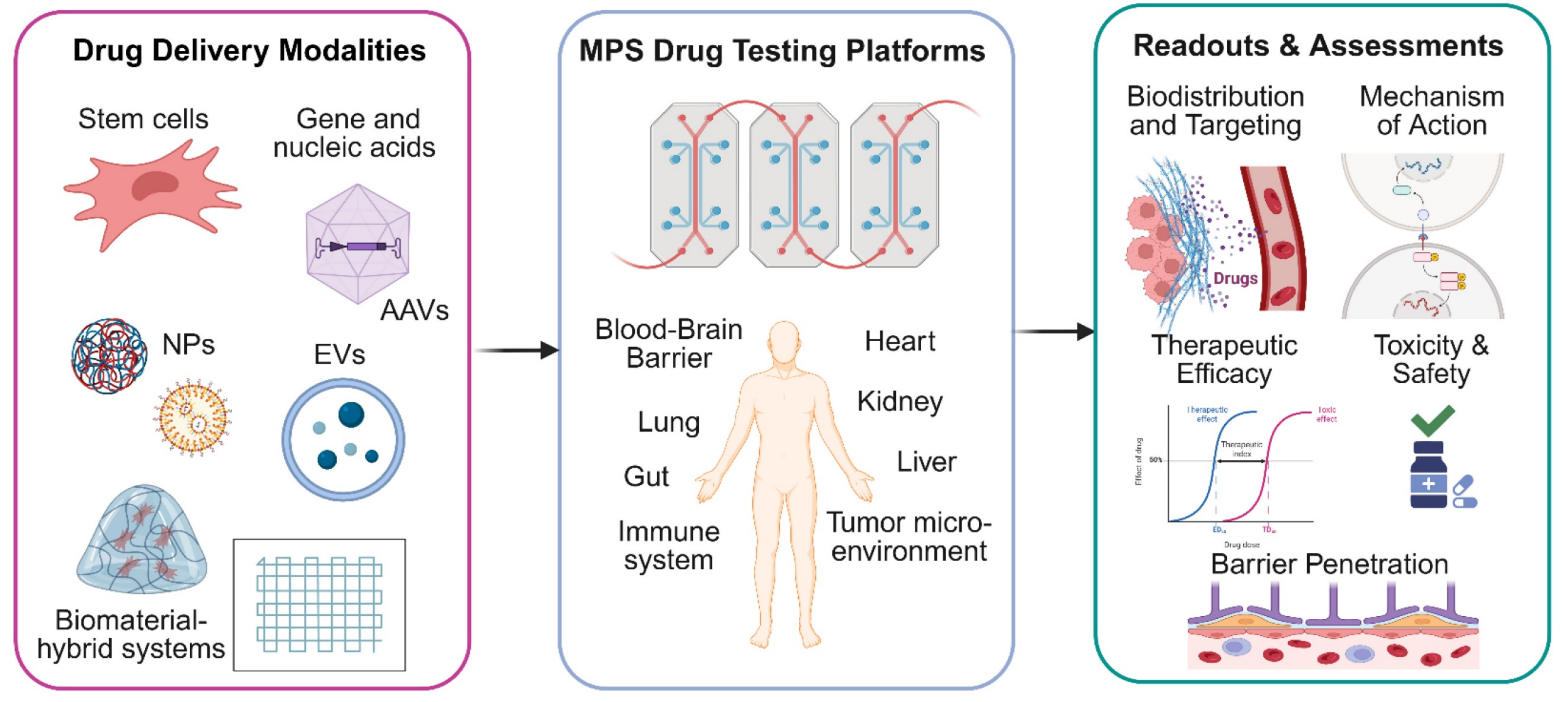
** Microphysiological systems (MPS) for evaluating next generation drug delivery strategies.** This schematic summarizes the application of multi-organ-on-a-chip (MOoC) platforms as advanced preclinical models for the evaluation of novel drug delivery modalities. Diverse therapeutic carriers, encompassing stem cell-based system, gene and nucleic acid therapies, engineered nanoparticles (NPs), extracellular vesicles (EVs) and biomaterial-hybrid systems are introduced into these integrated microphysiological platforms. The MOoC systems incorporate human-relevant organ and organ-system models, such as tumor, blood-brain barrier (BBB), liver, kidney, lung, gut, and cardiac tissues, interconnected through dynamic microfluidic circulation. This integrated approach facilitated the assessment of crucial parameters: (1) Biodistribution and Organ-Specific Accumulation of the therapeutic agent; (2) Barrier Penetration across physiological interfaces like the BBB; (3) Therapeutic Efficacy at target tissues (e.g., tumors); (4) Off-Target Toxicity and (5) Elucidation of Mechanisms of Action. Created in BioRender (https://BioRender.com/xdj22lo).

Compared with 2D cultures, MPS platforms incorporate flow conditions, vascular architecture tissue barriers, all of which influence nanoparticle margination, extravasation and tissue penetration. For example, tumor-on-a-chip systems with perfused microvasculature enable systematic tuning of nanoparticle size, shape, and surface chemistry while directly measuring intratumoral transport and therapeutic response 138-140. MPS platforms are also powerful systems for interrogating nanoparticle biodistribution, metabolism, clearance, and off-target toxicity, and therefore represent valuable tools for de-risking nanoparticle-based therapeutics prior to clinical translation (Figure 4).

### 3.4 Extracellular vesicle-mediated delivery in MPS

Extracellular vesicles (EVs), including exosomes and microvesicles, are increasingly recognized as natural nanocarriers due to their intrinsic biocompatibility, ability to cross biological barriers, and suitability for precision drug delivery 144-146. EVs can be derived from diverse cell types, including MSCs and engineered producer lines, and can be loaded with therapeutic RNAs, proteins, or genome-editing cargos. Current strategies focus on enhancing EV targeting specificity via ligand engineering, scaling up production, and implementing standardized purification protocols 147.

MPS enables the creation of physiologically relevant disease models where EV efficacy can be evaluated. For instance, Nguyen et al. developed a human kidney-on-a-chip to model acute kidney injury, demonstrating that MSC-derived EVs can recover the renal epithelium after injury 98. Similarly, Saferzadeh et al. used a complex fetal-maternal interface organ-on-a-chip to model infection-induced inflammation and showed that delivery of IL-10 via engineered EVs reduced inflammation associated with preterm birth 148. In addition to single-organ applications, MOoC platforms are well-suited for studying extracellular vesicle (EV) biodistribution and organ tropism. Kim et al. proposed a modular MOoC system to investigate EV-mediated interactions within the gut-brain-axis (GBA) 149. This system features a main body chip that houses multiple GBA organ modules, and its integrated circulation route connects each element, allowing for the observation of labeled EV delivery 149.

Because EVs interact with multiple cell types and barriers, MOoC platforms are particularly powerful for mapping their organ tropism and systemic effects. By integrating relevant organ modules under shared circulation, these systems can reveal how EV dosing and tissue-specific uptake can translate into therapeutic benefit. Critically important in the field of drug development, MPS systems also allow for investigation into EV drug delivered mechanisms of action. For example, Safarzadeh et al. leveraged their platform to show that their IL-10 therapy worked by activating IL-10 signaling pathways while inhibiting pro-inflammatory NF-kB activation 148. These systems also serve as robust preclinical platforms for assessing pharmacokinetics and safety, as demonstrated by the same study, which tracked EV propagation across a multi-layer biological barrier and confirmed the therapy's lack of cytotoxicity. Collectively, these studies highlight the utility of MPSs to accelerate the translation of EV therapeutics by providing real-time evaluation of biodistribution, immune modulation, and therapeutic outcomes across multiple tissue compartments, thereby accelerating clinical translation (Table 3) 150-152.

### 3.5 Biomaterial and hybrid delivery strategies in MPS

Biomaterials such as hydrogels, scaffolds, and synthetic extracellular matrix analogues provide spatiotemporal control of therapeutic release while supporting cell viability and tissue integration 153. These materials can be dynamically tuned to mimic native biochemical and mechanical cues, thereby enabling more predictive preclinical testing 154,155. Integration of biomaterial-based drug depots into MPS architectures further allows simulation of physiologically relevant pharmacokinetics and localized therapeutic action (Table 3) 156. Drug delivery systems can also achieve spatiotemporal control of payload release external triggers such as light or ultrasound. For example, thermoplastic polymeric particles can be constructed to release drugs upon a light trigger 157 and elastic eccentric microcapsules can have triggered release following an ultrasound trigger 158. These approaches can penetrate through the length scales of many MPS constructs. Another intriguing method of spatiotemporal control of biomolecules in a MPS device can be enabled using genetic cellular design combined with an external trigger. For example, Gordana Vunjak-Novakovic and coworkers recently demonstrated the ability to tune endothelial barrier permeability via light-stimulated RhoA signaling in an optogenetic organ-on-a-chip platform 159.

Compelling examples of spatiotemporal control of drug delivery in MPSs include the development of hybrid systems for localized cancer therapy. Chehri et al utilized a 'GlioMesh,' which combined a 3D-printed alginate hydrogel scaffold with drug-loaded PLGA microparticles 160. This hybrid biomaterial provided spatiotemporal control over the release of two different chemotherapeutics. Crucially, its efficacy was evaluated within a bespoke MPS containing 3D glioblastoma tumoroids embedded in a collagen matrix. This integrated 'tumoroid-on-a-plate' platform enabled the researchers to simultaneously evaluate the therapeutic efficacy and anti-invasive effects of the localized drug depot in a context that mimics the native tumor microenvironment, paving the way for more predictive, personalized therapeutic design 160. Hybrid systems that combine nanoparticles, EVs, and stem cell carriers with biomaterial scaffolds are emerging as cutting-edge approaches. In multi-organ MPS platforms, such hybrid strategies enable simultaneous evaluation of efficacy, toxicity, and off-target effects in interconnected organ systems, paving the way for personalized therapeutic design 11,161.

Biomaterial depots and hybrid constructs often exhibit complex, spatiotemporal controlled release profiles that interact with local tissue architecture. Embedding these biomaterial systems into MPS and MOoC models allows for measurement of release kinetics, gradient formation, and therapeutic impact into 3D human-relevant microenvironments. In addition, they can help capture how locally released drugs distribute to distal organs and whether degradation of biomaterials can induce off-target toxicity. Indeed, MPS systems can help provide a human-centric comprehensive view of safety and efficacy difficult to achieve with traditional models.

### 3.6 Immune system and lymphatic transport modeling in MPS

MOoC platforms that simultaneously integrate immune components, such as the mononuclear phagocyte system cells, and lymphatic structures to evaluate next-generation drug delivery systems under immune-mediated clearance and lymphatic transport conditions are not yet well established. Existing studies instead address isolated aspects of these questions, often using murine rather than human cells and tissues, typically in single-organ or lymph node focused MPS. As a result, there is currently no chip-based model that comprehensively links NP or EV design to immune activation, clearance and lymphatic routing within a unified MOoC framework.

Despite this limitation, recent single-organ lymphatic models have begun to elucidate how NPs interact with lymphatic endothelial cells (LECs). Lu et al. developed a lymphatics-on-a-chip model incorporating an engineered lymphatic vessel that drains interstitial fluid containing PLGA-b-PEG NPs of defined sizes. In this system, smaller particles (30 and 50 nm) diffused more rapidly through the interstitial matrix and were preferentially internalized and retained by LECs, paradoxically slowing their appearance in the lymphatic lumen compared with larger 70 nm particles 162. Through pharmacologic inhibition, Lu et al. further showed that dynamin-, caveolin-, and macropinocytosis-mediated endocytosis, together with regulation of endothelial junctions, are critical determinants of this size-dependent lymphatic transport 162.

Lymph-node-on-a-chip (LNOC) models have primarily been used to probe immune cell behavior and tissue-like architecture, with only limited, non-systematic exploration of carrier design. German et al. introduced a polydimethylsiloxane (PDMS)-based LNOC incorporating a freeze-cast collagen sponge supporting a 3D 4T1 tumor spheroid and examined penetration of fluorescent bovine serum albumin (BSA)-tannic-acid (TA) capsules (0.3 - 4 µm) delivered with lymphocytes, finding the smallest capsules penetrated more effectively into the spheroid 163. In addition, Cook et al. developed a 3D-printed, multi-compartment-on-a-chip platform that connects an upstream agarose “mock injection site” to a downstream slice of murine skin-draining lymph node tissue under recirculating flow 164. After introducing a model vaccine (Rho-OVA/R848) into the mock injection site, antigen drainage and spatial accumulation within the mouse LN slice was observed. The resulting patterns of antigen distribution and early activation marker expression resembled those seen after vaccination in mice 164. Complementing these murine tissue-based systems, Hallfors et al. developed a multi-compartment human LNOC supporting co-cultured Jurkat T cells and Raji B cells under continuous flow, allowing real-time analysis of lymphocyte motility and viability 165.

Together, these studies highlight that current MOoC platforms are still far from capturing the complexity of how innate and adaptive immune mechanisms, lymphatic transport, and clearance physiology influence the pharmacokinetics, biodistribution, and efficacy of next-generation drug modalities. The integration of human lymphatic endothelium, lymph node-like structures, and MPS organs into multi-organ, immune-competent systems thus remains an important and largely unmet goal for the field and represents a key opportunity to make MOoC platforms more predictive for immune-sensitive delivery strategies.

## 4. Current challenges and future perspectives

Multi-organ-on-a-chip platforms represent a transformative convergence of tissue engineering, microfluidics, and pharmaceutical sciences, offering unprecedented opportunities to bridge the translational gap between preclinical models and clinical outcomes. By recapitulating organ-level functionality, inter-organ crosstalk, and systemic pharmacokinetic behavior within physiologically scaled microenvironments, MOoCs address critical limitations of conventional *in vitro* assays and animal models 166. As demonstrated throughout this review, these systems have already enabled mechanistic insights into diverse drug delivery modalities, from viral vectors and nanoparticles to extracellular vesicles and cell-based therapies, while revealing delivery barriers and toxicity profiles that would be difficult to predict using traditional approaches. Looking forward, a variety of biological and technical challenges, along with regulatory advances define the landscape for future progress within the field. Here we highlight several of these challenges.

Biological limitations include cell sourcing; as iPSCs, though desirable for the ability to create patient-specific and diverse cell cultures, face challenges with reproducibility across donors and experiments and often exhibit incomplete maturation during extended culture 17,21,167. Emerging use of isogenic and genome-edited iPSC lines offers partial solutions by improving batch consistency and enabling controlled genotype-phenotype comparisons 33,34. The lack of immune cell components also poses a significant gap in understanding of a major part of clinical evaluation that current MOoC models fail to capture 168.

Pharmacological limitations highlight PK/PD modeling difficulties, due to the issue of scaling chip models and organ-to-organ ratios to physiological values. Also, there is a significant bias towards small-molecule studies with MOoC platforms. While much progress has been made in terms of MOoC models for examining small-molecule drug delivery, many other modalities have yet to be thoroughly examined using this new technology. Larger biologics and peptides, gene therapies, and nanoparticles have all been tested on single-organ chip models, but a significant gap lies in holistically examining their pharmacokinetic and pharmacodynamic properties across multiple relevant tissues under shared, physiologically relevant flow conditions 169. As stated in section 2.8 of this review, computational modeling and digital-twin frameworks are beginning to emerge as tools to bridge this gap, linking experimental chip data with *in silic*o PK/PD scaling for whole-body predictions 9.

As opposed to most small molecules, biologics and peptides are often too large to undergo passive diffusion and therefore rely on mechanisms such as receptor-mediated endocytosis, particularly at restrictive barriers such as the blood-brain barrier 36. In this context, the inclusion of sequential organ modules such as gut, liver, and BBB within a single MOoC could capture an entire sequence of regions of loss along the path to CNS exposure. The development of endothelial barriers in multi-organ models offers a mode of tracking how these larger molecules pass through each barrier in a sequential manner.

Gene therapies and nucleic acid-based therapeutics face a different major hurdle, namely reaching specific disease-relevant cells while avoiding degradation and expression in off-target sites. Traditional *in vitro* cultures and animal modeling often lack the reproduction of human-specific biodistribution, clearance, and immune responses needed for a holistic understanding of vector fate. A paper by Achberger et al. has shown the use of a retina-on-chip model for validating adeno-associated virus (AAV) retinal gene therapy vectors. They demonstrated that their model accurately distinguished capsid variants under controlled microfluidic perfusion and retained retinal tissue viability for long-term monitoring 129. Further development into a multi-organ model could enable mimicking vector delivery through systemic circulation. This model, with further tissue complexity, would be important because these interactions can significantly affect efficacy in patients. Perhaps most importantly, the authors identify that a major shortcoming of their single-organ model is the lack of immune components in their system, resulting in an inability to assess immune responses to AAV vectors. This could be enabled through a multi-organ model incorporating immune cell populations. Future directions may also include coupling vector biodistribution assays with liver, spleen, and kidney modules to capture clearance and innate-immune recognition dynamics 126,130.

For nanoparticles, future MOoCs could be designed to focus on understanding biodistribution, passing through clearance organs such as the liver, before reaching their target. Studies have emphasized that the development of nanomaterials, while becoming quite popular in clinical applications, is significantly delayed due to the inability to test efficacy 7. The integration of multiple tissue types together within the same MPS, including especially macrophages and immune system phagocytosis, kidney filtration and clearance, and potential hepatotoxicity of degradation products, are all key to modeling the many delivery challenges faced by next-generation therapeutics. With the many possibilities offered by MOoCs, these platforms can help the field move away from animal testing toward improved, clinically relevant evaluation of particle-based therapeutics at lower cost and higher throughput.

A fundamental limitation of current MOoC platforms is the inability to fully recapitulate functional circulatory networks that are essential for physiologically accurate drug distribution and excretion. Although vascularized organ-on-chip models have advanced considerably, many existing systems rely on simplified endothelial-lined channels or non-perfusable capillary networks that lack the hierarchical branching, flow dynamics, and barrier properties of native vasculature 170,171. True perfusable microvascular networks that approximate physiological vessel diameters, branching architecture, and appropriate shear stress profiles remain technically challenging to engineer and maintain long-term, particularly when integrated across multiple organ modules 171,172. The lymphatic system, which plays critical roles in fluid homeostasis, immune cell trafficking, and drug clearance, is even more underrepresented in MOoC designs. Recent proof-of-concept studies have demonstrated lymphatic endothelial cell integration into single-organ chips and shown differential biomarker expression under lymphatic versus blood flow condition 173, however developed lymphatic drainage systems connected to blood vasculature within multi-organ platforms are virtually nonexistent. This absence prevents accurate modeling of the role of lymphatic clearance in drug pharmacokinetics, particularly for biologics and nanoparticles that preferentially drain through lymphatics.

There are still several technical limitations being considered in the development of MOoCs. PDMS, a polymer often utilized in chip fabrication, often causes significant, non-specific absorption and adsorption of small, hydrophobic drug molecules. This phenomenon causes substantial loss of drug concentration, sometimes over 90%, leading to inaccurate dose-response results, impaired translatability, and incorrect pharmacological data 174. Quantitative studies have established that absorption is strongly correlated with a compound's partition coefficient, with molecules exceeding a logP threshold of approximately 2.5 being particularly susceptible, though parameters such as topological polar surface area and exposure time further complicate predictions. Finite element modeling approaches have since been employed to simulate drug concentration dynamics within PDMS devices, enabling more accurate dose-response corrections without requiring full material substitution 175,176. This has prompted exploration of alternative polymers or surface coatings 5,8. Recent efforts have focused on replacing PDMS with cyclic olefin copolymers (COC/COP), thiol-ene polymers, and high-resolution 3D-printed photopolymers that minimize drug sorption and improve optical and mechanical stability 6,16. A recent study by Mair et al. shows that utilizing a PDMS-PEG copolymer, and pretreatment of the polymer with the drug prior to experimental testing can significantly reduce drug absorption 177. Additionally, new modes of perfusion are being developed to attain more physiologically accurate flow systems for long-term culture, with the goal of maintaining stable shear stress profiles and minimizing media loss or contamination over extended studies 14,16. Parallel advances in automated plate-based and pneumatic circulation systems have begun to improve throughput, yet reproducibility and cross-site standardization remain major obstacles 16.

From a fabrication standpoint, producing MOoC systems presents substantially greater challenges compared to single-organ platforms. While soft lithography remains a predominant technique, MOoC manufacturing requires intricate multi-step lithographic processes, precise alignment of multiple layers, and integration of distinct organ compartments with controlled geometries and volume ratios 178. These alignment and bonding steps become increasingly labor-intensive and error-prone as system complexity increases, with device yields often compromised by fabrication defects or fluidic leakage. Fluidic interconnection is also a key challenge, as connecting multiple organ modules via tubing, pumps, and reservoirs introduces dead volumes, increases contamination risk, and requires robust long-term sealing strategies 179,180. Bubble formation within microfluidic channels can occlude flow and cause experimental failure, an issue exacerbated in multi-compartment systems 179. Bioprinting has emerged as a promising alternative for constructing tissue-laden MOoC platforms, but still presents limitations, including limited resolution compared to photolithography, challenges in post-printing device sealing and assembly, difficulties in achieving vascularization within printed constructs, and the need for bioinks that maintain cell viability across all printing stages. Additionally, integrating bioprinted tissues into closed-loop microfluidic systems remains technically demanding, as printed constructs must withstand shear stress induced by perfusion, while maintaining structural integrity over culture duration 181.

In parallel with technological advances, the regulatory and policy landscape surrounding MPS and MOoC platforms has evolved rapidly. The passage of the U.S. FDA Modernization Act 2.0 formally acknowledged New Approach Methodologies (NAMs), including organ-on-chip technologies, as potential contributors to drug development and regulatory decision-making 182. Also, the European Medicines Agency (EMA) have emphasized the potential value of MPS platforms for evaluating human-specific metabolism, toxicity, and complex modalities such as biologics, gene therapies, and nanoparticle-based therapeutics, while underscoring the importance of context-specific validation 183. This shift reflects increasing recognition that human-relevant *in vitro* systems can complement traditional animal models, particularly in areas where animal-to-human translation is limited 184. Importantly, regulatory agencies do not currently position MOoC platforms as direct replacements for animal testing, but rather as complementary, decision-support tools that can improve mechanistic understanding, enable early de-risking, and inform candidate selection and study design 185.

Concurrently, significant efforts are underway to establish technical standards and best practices for MPS and MOoC development 186,187. International organizations and consortia, including the OECD, ASTM International, and the IQ MPS Affiliate, are actively developing guidelines related to device characterization, reproducibility, quality control, and data reporting. These standardization initiatives aim to enhance cross-platform comparability and experimental robustness, which are essential prerequisites for broader industrial adoption and regulatory confidence. Together, evolving regulatory engagement and standardization efforts signal a transition of MOoC platforms from exploratory research tools toward increasingly translational technologies. Continued dialogue among technology developers, end users, and regulatory agencies will be critical for defining appropriate contexts of use, validation benchmarks, and pathways for integrating MOoC-derived data into pharmaceutical development pipelines.

## Figures and Tables

**Table 1:** Engineering advances enabling MOoC development

Engineering Feature	Technology/Approach	Advantages	Key Applications	Representative Systems	References
Perfusion Systems	Pumpless gravity-driven flow	No external pumps, reduced complexity, inter-well pressure gradients	Long-term culture (14+ days), reduced contamination	Multi-organ PK-PD platform	13
	Closed-loop circulation	Minimal media loss, reduced tubing	28-day liver-skin co-culture	Dynamic MOoC	14
	Pneumatic pressure control	Programmable flow, centralized control	High-throughput screening	96-well pneumatic platform	16
Scaling & Throughput	96-well plate format	Standard laboratory equipment, automated handling	Drug screening, toxicology testing	Dual perfusion bioreactor	15
	Modular compartments	Independent organ control, flexible configuration	Custom organ pairing, disease modeling	Various gut-brain designs	17,19,20
	Systemic multi-organ integration	Holistic inter-organ communication modeling	Disease pathways, drug metabolism	Multi-organ systemic platforms	9
Barrier Engineering	Permeable membranes with tight junctions	Physiological permeability, selective transport	BBB, gut epithelium, kidney tubules	NVU chips, renal barriers	31
	Dual-flow systems	Separate apical/basolateral perfusion	Polarized epithelial function	96-well renal platform	15
	3D self-organized barriers	Physiological microvessel architecture	BBB modeling with realistic structure	Microvascular BBB models	25
Biomaterials	Synthetic hydrogels	3D tissue constructs, tunable ECM properties	Tissue maturation, cell viability	3-tissue organ platform	12,153
	Bioprinting	Organ-specific ECM, spatial control	Custom tissue architecture	Various applications	12,153,154
	Biomaterial-drug depots	Spatiotemporal drug release control	Localized therapeutic action	Hydrogel mesh for glioblastoma, shape memory polymers	156,157,160
	Tumor modeling biomaterials	Biomimetic tumor microenvironment	Cancer drug testing, metastasis studies	Tumor-on-chip platforms	155
Monitoring & Sensing	Integrated biosensors	Real-time readouts, continuous monitoring	TEER, oxygen, metabolites	Gut-brain donepezil study, BBB-on-chip	20,38
	Multi-electrode arrays (MEA)	Electrophysiological monitoring, functional assessment	Cardiac and neural activity, drug-induced effects	Heart-on-chip, neuromuscular systems	84,86,87
	Microplate readers	Automated imaging, high-content analysis	Throughput screening	96-well systems	15,16
	Integrated bioelectronics	Extra- and intracellular monitoring	Cardiac electrophysiology under stress	Heart-on-chip with hypoxia	86
Advanced Integration	Optogenetic control	Dynamic barrier modulation, temporal control	Endothelial barrier studies, neuromuscular actuation	OptoBarrier platform, optogenetic neurons	60,159
	AI integration	Enhanced predictive modeling, data analysis	Future drug screening platforms	Cancer modeling	161
	Patient-specific iPSC systems	Personalized disease modeling, precision medicine	Patient-specific drug screening	BBB chips, cardiac models	33,75
Validation & Maturity	Standardization approaches	Reproducibility, quality control	Regulatory acceptance, industrial adoption	MPS as reliable tools	10,131
	Biological product assessment	Testing of biologics, new modalities	Therapeutic antibodies, cell therapies	Advanced MPS platforms	126
	New drug modality testing	Toxicity assessment beyond small molecules	Gene therapies, biologics, complex formulations	Comprehensive MPS systems	11,169

**Table 2:** Comparison of representative MOoC platform designs

Platform Type	Organs/Tissues Included	Perfusion Method	Culture Duration	Key Readouts	Reference
4-Organ Chip	Intestine, liver, skin, kidney	Recirculating medium	28 days	CYP activity, barrier integrity, metabolite distribution	4
3-Tissue Platform	Liver, cardiac, skeletal muscle	Integrated microfluidics	14+ days	Multi-tissue interactions, drug metabolism	12
Gut-Brain Axis	Gut epithelium (Caco-2/iPSC), BBB (hBMECs, astrocytes, pericytes)	Dual compartment with independent flow	7-14 days	TEER, permeability, cytokine panels, neurotransmitter levels, EV-mediated communication	19,20,149
Neurovascular Unit	Endothelial cells, pericytes, astrocytes, neurons	Dynamic perfusion with shear stress	14-21 days	Tight junction integrity, transporter expression, electrophysiology, stem cell therapy evaluation	32
Liver-Kidney	Hepatocytes, proximal tubule epithelium	Microfluidic circulation	14-28 days	Transporter activity (OAT, OCT), metabolite clearance, albumin production, EV biodistribution	97,98
Lung-Liver	Alveolar epithelium, hepatocytes	Pneumatic microfluidics	7-14 days	Cytokine production, barrier function, metabolic conversion, inflammatory crosstalk	94
Heart-Liver	Cardiac tissue, hepatocytes	Microfluidic perfusion	14+ days	Hepatic metabolism effects on cardiotoxicity, metabolite-induced cardiac effects	92
Heart-Liver-Skin	Cardiac, hepatic, skin	Microphysiological perfusion	7-14 days	Topical drug delivery, systemic distribution, organ-specific responses	79
Multi-organ (Heart-Liver-Tumor-Lung)	Cardiac, hepatic, tumor spheroids, lung	Pumpless gravity-driven	14 days	Drug efficacy, off-target toxicity, PK/PD modeling	95
Cardiovascular-Kidney-Metabolic	Heart, kidney, metabolic tissues	Integrated circulation	Variable	Interorgan crosstalk, CKM syndrome modeling	93
Heart-Brain Codevelopoid	Cardiac and neural tissues via codevelopment	Trans-germ-layer integration	Extended culture	Heart-brain axis interactions, codevelopment signaling	80
iPSC-derived Personalized BBB	Patient-specific endothelial, pericytes, astrocytes	Perfused microfluidic	7-14 days	Disease modeling (AD, PD), personalized drug screening	33

**Table 3:** Drug delivery modalities tested in MOoC systems

Delivery Strategy	Specific Examples	MOoC Platform Used	Key Findings	Current Limitations	References
Stem Cell-Based	MSCs for immunomodulation, NSCs for CNS delivery, engineered stem cells	NVU-on-chip, ischemic stroke model, various organ chips	MSCs restored BBB integrity and neuronal survival post-ischemia; stem cells as delivery vehicles demonstrated	Immune rejection risk, differentiation control, limited multi-organ studies, scalability	31,113,116-118,173
Gene Therapy	AAV vectors for retinal delivery, CRISPR/Cas9 delivery, gene transfer nanoparticles	Retina-on-chip (single organ), various MPS platforms	Capsid variant screening, long-term viability, improved targeting and efficiency	Lacks immune components in most models; needs multi-organ integration for systemic delivery; off-target effects	121,128-130
Nanoparticles	Polymeric NPs, lipid NPs, metal NPs, functionalized nanorods, nanoshuttles	Tumor-on-chip, BBB-on-chip, liver-kidney systems, tumor microenvironment chips	Biodistribution tracking, clearance pathways, toxicity assessment, BBB penetration strategies, tumor-specific delivery	Limited systematic multi-organ PK/PD studies; need for better predictive *in vivo* correlation	7,127,133-135,138-140,142,143
Extracellular Vesicles	MSC-derived EVs, engineered exosomes, iPSC-organoid-derived EVs	Liver-kidney organoid MOoC, lung injury chip, gut-brain axis, feto-maternal interface, cardiac regeneration models	Biodistribution altered by liver dysfunction; therapeutic effects in acute lung injury; gut-brain communication; pregnancy pathology modeling	Scale-up production, standardization challenges, loading efficiency, targeting specificity	98,144,147-151
Biomaterials	Hydrogels, scaffold-based depots, shape memory polymers, drug-eluting meshes, microcapsules, hybrid systems	3-tissue platform, wound repair models, glioblastoma models, tumor models	Sustained release, spatiotemporal control, tissue integration, localized drug delivery, controlled release mechanisms	Limited representation in current MOoC literature; need for more multi-organ validation	12,153-158,160
Small Molecules	Chemotherapeutics, targeted drugs, donepezil, antidepressants, topical agents	Heart-liver-tumor-lung, gut-brain axis, multi-organoid systems	Metabolic conversion, organ-specific toxicity, PK/PD correlation, off-target effects, safety assessment	Most common modality; well-studied but still gaps in complex multi-organ interactions	20,78,79,94,95
